# Sphingosine kinase 1 is a reliable prognostic factor and a novel therapeutic target for uterine cervical cancer

**DOI:** 10.18632/oncotarget.4818

**Published:** 2015-07-28

**Authors:** Hyun-Soo Kim, Gun Yoon, Ji-Yoon Ryu, Young-Jae Cho, Jung-Joo Choi, Yoo-Young Lee, Tae-Joong Kim, Chel-Hun Choi, Sang Yong Song, Byoung-Gie Kim, Duk-Soo Bae, Jeong-Won Lee

**Affiliations:** ^1^ Department of Pathology, Severance Hospital, Yonsei University College of Medicine, Seoul, Republic of Korea; ^2^ Department of Obstetrics and Gynecology, Pusan National University Yangsan Hospital, Pusan National University School of Medicine, Yangsan-si, Gyeongsangnam-do, Republic of Korea; ^3^ Department of Obstetrics and Gynecology, Samsung Medical Center, Sungkyunkwan University School of Medicine, Seoul, Republic of Korea; ^4^ Department of Pathology and Translational Genomics, Samsung Medical Center, Sungkyunkwan University School of Medicine, Seoul, Republic of Korea; ^5^ Department of Health Sciences and Technology, Samsung Advanced Institute for Health Science and Technology, Sungkyunkwan University, Seoul, Republic of Korea

**Keywords:** Pathology Section, sphingosine kinase 1, cervical cancer, progression, prognosis, FTY720

## Abstract

Sphingosine kinase 1 (SPHK1), an oncogenic kinase, has previously been found to be upregulated in various types of human malignancy and to play a crucial role in tumor development and progression. Although SPHK1 has gained increasing prominence as an important enzyme in cancer biology, its potential as a predictive biomarker and a therapeutic target in cervical cancer remains unknown. SPHK1 expression was examined in 287 formalin-fixed, paraffin-embedded cervical cancer tissues using immunohistochemistry, and its clinical implications and prognostic significance were analyzed. Cervical cancer cell lines including HeLa and SiHa were treated with the SPHK inhibitors SKI-II or FTY720, and effects on cell survival, apoptosis, angiogenesis, and invasion were examined. Moreover, the effects of FTY720 on tumor growth were evaluated using a patient-derived xenograft (PDX) model of cervical cancer. Immunohistochemical analysis revealed that expression of SPHK1 was significantly increased in cervical cancer compared with normal tissues. SPHK1 expression was significantly associated with tumor size, invasion depth, FIGO stage, lymph node metastasis, and lymphovascular invasion. Patients with high SPHK1 expression had lower overall survival and recurrence-free survival rates than those with low expression. Treatment with SPHK inhibitors significantly reduced viability and increased apoptosis in cervical cancer cells. Furthermore, FTY720 significantly decreased *in vivo* tumor weight in the PDX model of cervical cancer. We provide the first convincing evidence that SPHK1 is involved in tumor development and progression of cervical cancer. Our data suggest that SPHK1 might be a potential prognostic marker and therapeutic target for the treatment of cervical cancer.

## INTRODUCTION

Cervical cancer is one of the most common malignant neoplasms worldwide, and is responsible for the death of thousands of women every year [1, 2]. Although there is increasing evidence that early detection of cervical cancer has reduced mortality, the prognosis of advanced or recurrent cervical cancer remains very poor. Current treatment for cervical cancer, including surgery with radiation therapy, chemotherapy, and concurrent chemoradiation therapy, can yield cures in 80% to 90% of women with early stage I and II cervical cancer and 60% of those with stage II disease. In contrast, effective treatment options for advanced or recurrent cervical cancer are exceedingly limited. Cisplatin-based combination chemotherapy, the most commonly used cytotoxic therapy [3], has yielded response rates ranging from 20% to 30% and overall survival of less than 10 months in these patients [4]. Because of the minimal degree of success with cytotoxic therapies and the poor prognosis of patients, there is increased interest in targeted therapeutics for the management of advanced, recurrent, or metastatic cervical cancer.

An emerging area of investigation in lipid research has been the role of sphingolipids in cancer biology. Many groups are actively investigating the role of different sphingolipid enzymes, sphingolipid binding proteins, and transmembrane transporters in human cancers [5]. Among these, members of the sphingosine kinase (SPHK) family have received the most attention as important enzymes in cancer biology because their catalytic activity lies at a critical intersection in the regulation of bioactive sphingolipid metabolism. SPHK exists as two isoforms: SPHK1 and SPHK2. Sphingosine 1-phosphate (S1P), one of the metabolites, plays a crucial role in intracellular and extracellular signaling [6]; SPHK1 catalyzes the phosphorylation of sphingosine to form S1P, which regulates multiple cellular processes including inhibition of apoptosis, increased cell proliferation, and angiogenesis [7]. Accumulating evidence has suggested that SPHK1 is involved in processes associated with cancer progression, including cell transformation, survival, and migration, metastasis, and tumor microenvironment neovascularization [8].

SPHK1 is upregulated in human cancers of many different organs including head and neck [6], stomach [7], lung [9], brain [10, 11], colon [12, 13], and ovary [14]. In this regard, SPHK1 may be a suitable therapeutic target, and inhibitors of SPHK1 have been proposed as potential anticancer agents [15, 16]. For example, silencing the expression of SPHK1 in glioblastoma cells and breast cancer cells induced cell cycle arrest [17], and inhibition of SPHK1 dramatically decreased breast cancer formation [18]. Blocking SPHK1 activity suppressed tumor growth and reduced tumor occurrence and metastasis in nude mice [19, 20]. Similarly, we recently demonstrated that treatment with SPHK inhibitors significantly reduced cell proliferation, angiogenesis, and invasion, and increased apoptosis in ovarian cancer cells [16]. However, there are no available data on the expression of SPHK1 in cervical cancer and its biological and clinical significance. Furthermore, the therapeutic effects of SPHK inhibitors in cervical cancer remain unknown. The purpose of this study was to evaluate the clinical implications and prognostic significance of SPHK1 expression and to investigate the *in vitro* and *in vivo* effects of targeting SPHK1 with pharmacological inhibitors in cervical cancer.

## RESULTS

### Protein expression of SPHK1 in human cervical cancer tissues and cell lines

**Figure 1:**
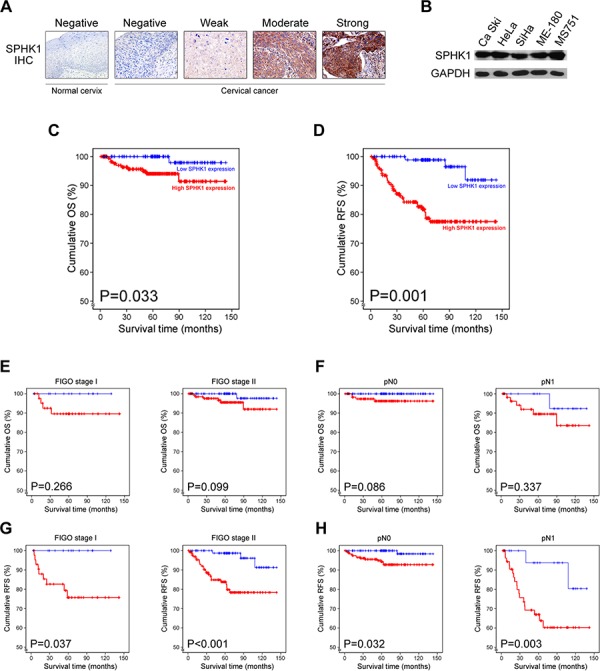
SPHK1 overexpression in human cervical cancer and its prognostic significance **A.** Immunohistochemical staining of SPHK1 in normal cervix and cervical cancer tissues. **B.** Western blot analysis of SPHK1 in cervical cancer cell lines. **C** and **D.** Kaplan-Meier curves showing (C) OS and (D) RFS of cervical cancer patients. **E** to **H.** Kaplan-Meier curves showing (E and F) OS and (G and H) RFS of the patient subgroups divided according to the status of FIGO stage and lymph node metastasis.

We examined SPHK1 protein expression in 287 cervical cancer and 5 normal cervical tissue samples using immunohistochemical staining. Representative photomicrographs of SPHK1 immunostaining are shown in Figure 1A. We observed that 63.8% (183/287) of the cancer tissue samples showed high SPHK1 expression and 36.2% (104/287) showed low expression. Immunoreactivity for SPHK1 was mainly localized in the cytoplasm of cancer cells, which is consistent with previous studies on SPHK1 expression in other types of human cancer [7, 10, 12, 16, 21, 22]. In contrast, none of the normal cervical tissue samples exhibited SPHK1 expression. Consistent with findings from the tissue samples, SPHK1 protein was strongly expressed in all human cervical cancer cell lines examined (Figure 1B).

### SPHK1 expression is significantly associated with aggressive oncogenic behavior of cervical cancer

We examined the relationship between SPHK1 expression in cervical cancer tissue and different clinicopathological characteristics (Table 1). High SPHK1 expression was associated with larger tumor size (*p* < 0.001), deeper invasion depth (*p* < 0.001), presence of lymph node metastasis (*p* = 0.029), higher FIGO stage (*p* = 0.029), presence of lymphovascular invasion (*p* = 0.045), and higher preoperative squamous cell carcinoma (SCC) antigen level (*p* = 0.009), indicating that SPHK1 expression is associated with aggressive oncogenic behavior of cervical cancer. However, no correlation was observed between SPHK1 expression and age, parametrial invasion, and vaginal resection margin involvement.

**Table 1:** Relationship between SPHK1 expression and clinicopathologic characteristics

Characteristics		Total	SPHK1 expression		*p* value
			High	Low	
Age (years)	≥49	141	87 (61.7)	54 (38.3)	0.475
	<49	146	96 (65.8)	50 (34.2)	
Tumor size (cm)	≥4.0	132	81 (80.2)	20 (19.8)	<0.001*
	<4.0	155	102 (54.8)	84 (45.2)	
Depth of invasion (cm)	≥1.0	159	124 (78.0)	35 (22.0)	<0.001*
	<1.0	128	59 (46.1)	69 (53.9)	
Lymph node metastasis	Present	77	57 (74.0)	20 (26.0)	0.029*
	Absent	210	126 (60.0)	84 (40.0)	
FIGO stage	IA	15	9 (60.0)	6 (22.4)	0.029*
	IB	214	129 (60.3)	85 (39.7)	
	II	58	45 (77.6)	13 (22.4)	
Lymphovascular invasion	Present	130	91 (70.0)	39 (30.0)	0.045*
	Absent	157	92 (58.6)	65 (41.4)	
Parametrial invasion	Present	33	23 (69.7)	10 (30.3)	0.451
	Absent	254	160 (63.0)	94 (37.0)	
Resection margin involvement	Present	14	10 (71.4)	4 (28.6)	0.777
	Absent	273	173 (63.4)	100 (36.6)	
Preoperative SCC (ng/mL)	≥1.5	137	98 (71.5)	39 (28.5)	0.009*
	<1.5	150	85 (56.7)	65 (43.3)	

* Statistically significant (*p* < 0.05).

### SPHK1 expression is an independent prognostic factor for recurrence-free survival in patients with cervical cancer

Adequate clinical follow-up information was available for all 287 patients with cervical cancer. The mean survival time for the patient cohort was 5.55 years. Death was reported in 11 of 287 cases (3.8%), of which 10 (90.9%) had high SPHK1 expression. As shown in Table 2, univariate analysis for OS revealed that SPHK1 expression (*p* = 0.033; Figure 1C), tumor size (*p* = 0.033), and lymph node metastasis (*p* = 0.008) were associated with OS. Multivariate analysis demonstrated that lymph node metastasis (*p* = 0.033) independently predicted OS. Although SPHK1 expression was found to have the highest relative risk for death (5.643), it did not predict OS by itself (*p* = 0.101).

**Table 2:** Univariate and multivariate analyses for OS and RFS

Characteristics		OS				RFS			
		Univariate	Multivariate			Univariate	Multivariate		
		*p* value	*p* value	RR	95% CI	*p* value	*p* value	RR	95% CI
Age (years)	≥49	0.732	NA			0.658	NA		
Tumor size (cm)	≥4.0	0.033*	0.396	1.785	0.468–6.815	0.009*	0.570	1.254	0.574–2.742
Depth of invasion (cm)	≥1.0	0.100	0.799	1.248	0.227–6.852	0.049*	0.442	0.715	0.304–1.683
Lymph node metastasis	Present	0.008*	0.033*	3.842	1.115–13.238	<0.001*	0.013*	2.483	1.208–5.106
FIGO stage	II	0.129	NA			0.128	NA		
Lymphovascular invasion	Present	0.066	0.498	1.668	0.380–7.326	<0.001*	0.022*	2.716	1.156–6.379
Parametrial invasion	Present	0.456	NA			0.099	0.449	1.398	0.587–3.328
Resection margin involvement	Present	0.533	NA			0.369	NA		
Preoperative SCC (ng/mL)	≥1.5	0.582	NA			0.063	0.677	0.848	0.392–1.838
SPHK1 expression	High	0.033*	0.101	5.643	0.714–44.590	<0.001*	0.008*	3.604	1.388–9.365

Abbreviations: OS, overall survival; RFS, recurrence-free survival; RR, relative risk; CI, confidence interval; NA, not applicable.

* Statistically significant.

Recurrences were reported in 35 of 287 cases (12.2%), and 91.4% (32/35) of these had high SPHK1 expression. Univariate analysis for RFS showed that SPHK1 expression (*p* = 0.008; Figure 1D), tumor size (*p* = 0.009), depth of invasion (*p* = 0.049), lymph node metastasis (*p* < 0.001), and lymphovascular invasion (*p* < 0.001) were significant predictors of poor RFS (Table 2). Furthermore, in multivariate analysis, SPHK1 expression, lymph node metastasis, and lymphovascular invasion were identified as independent prognostic factors for RFS. This analysis also demonstrated that high SPHK1 expression in cervical cancer had the highest relative risk of recurrence (3.604), superior to the risk associated with lymph node metastasis (2.483) or lymphovascular invasion (2.716).

Moreover, we evaluated the prognostic value of SPHK1 expression in selected patient subgroups. We did not find any difference in OS when patients with high and low SPHK1 expression were grouped according to the FIGO stage (Figure 1E) or presence of lymph node metastasis (Figure 1F). In contrast, there were significant differences in RFS between the high and low SPHK1 expression groups for FIGO stage I (*p* = 0.037) and stage II (*p* < 0.001; Figure 1G). In addition, there were significant differences between the RFS of patients with high expression and low expression according to the absence (*p* = 0.032) or presence (*p* = 0.003) of lymph node metastasis (Figure 1H). Taken together, our data suggest that SPHK1 is a potentially useful predictor for outcome of cervical cancer. In particular, we found that SPHK1 is a novel independent biomarker for predicting RFS of patients with cervical cancer.

### SPHK inhibitors significantly affect cell survival and apoptosis in cervical cancer cells

**Figure 2:**
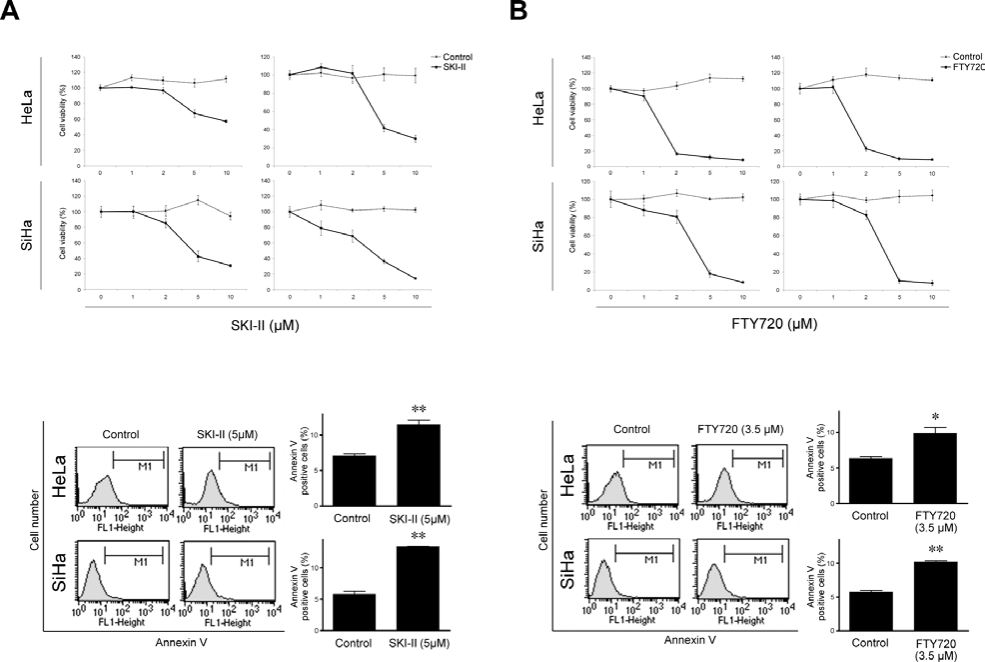
Effects of SPHK inhibitors SKI-II and FTY720 on cell survival and apoptosis in HeLa and SiHa cells Both **A.** SKI-II and **B.** FTY720 elicited cytotoxic effects (upper; MTT assay) and increased apoptosis (lower; FACS analysis). FTY720 exerted more potent inhibitory effects than SKI-II in both cell lines. The error bar represents standard error of mean. *p < 0.05, **p < 0.01.

We used two SPHK inhibitors, SKI-II and FTY720, to block the endogenous activity of SPHK1 in human cervical cancer cell lines. In both HeLa and SiHa cells, SKI-II significantly reduced cell viability in a dose-dependent manner and increased apoptosis (Figure 2A). FTY720 inhibited cervical cancer cell survival to a greater degree than SKI-II in both cell lines (Figure 2B).

### Effect of SPHK inhibitors on SPHK1 enzymatic activity and levels of MMP-2 and VEGF-A

**Figure 3:**
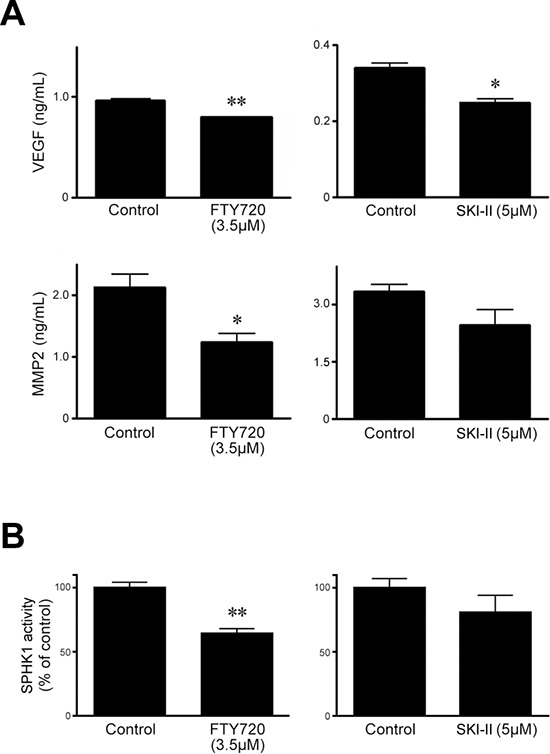
Effects of SPHK inhibitors SKI-II and FTY720 on levels of VEGF-A and MMP-2 and enzymatic activity of SPHK1 in HeLa cells **A.** The level of VEGF-A (upper), assessed by ELISA (24 hr) was significantly decreased after treatment with FTY720 or SKI-II. In contrast, MMP-2 level was significantly reduced only after treatment with FTY720. **B.** Similarly, FTY720, but not SKI-II, significantly decreased SPHK1 enzymatic activity. The error bar represents standard error of mean. *p < 0.05, **p < 0.01.

FTY720 significantly decreased the expression of both matrix metalloproteinase (MMP)-2 and vascular endothelial growth factor (VEGF)-A in HeLa cells whereas SKI-II significantly reduced the expression of VEGF-A, but not that of MMP-2 (Figure 3A). To confirm the specific effects of these inhibitors on SPHK1, we measured SPHK1 enzymatic activity. FTY720, but not SKI-II, significantly repressed the enzymatic activity (Figure 3B).

### FTY720 inhibits tumor growth in patient-derived xenograft cervical cancer model

**Figure 4:**
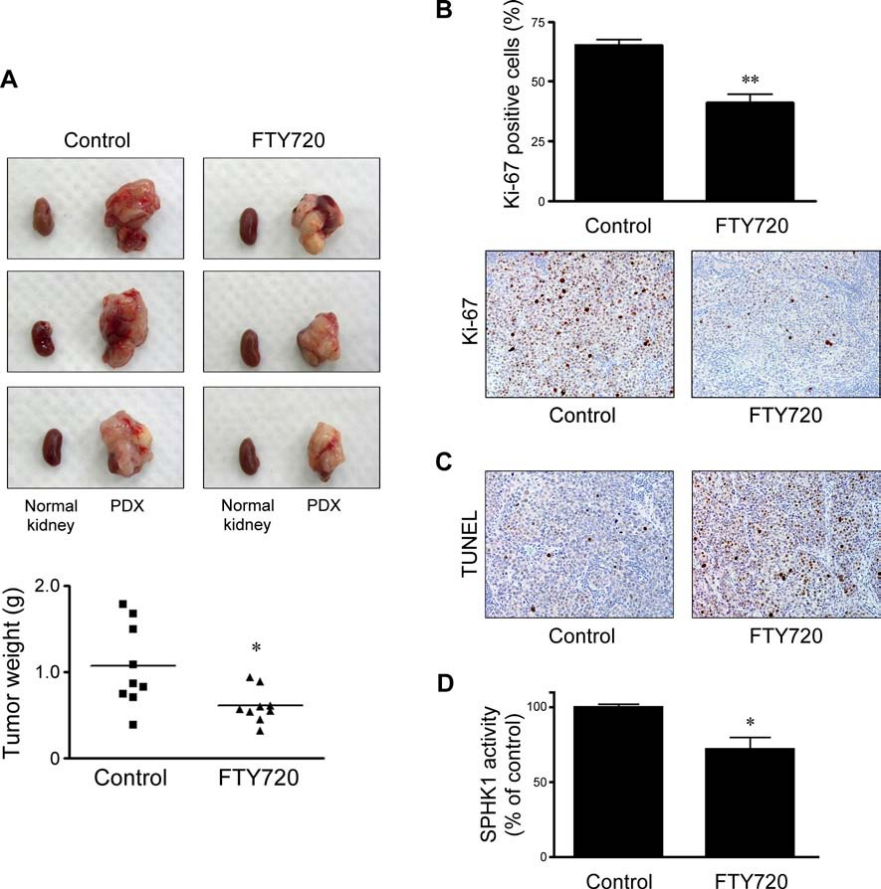
Effects of FTY720 on *in vivo* tumor weight, cell proliferation, apoptosis, and enzymatic activity of SPHK1 in cervical cancer PDX models **A.** In each image, normal kidney (left) and a developed xenograft tissue are shown. FTY720 resulted in a significantly reduced tumor weight compared with PBS-injected control. **B.** In the FTY720-treated group, harvested tumor tissues showed significantly decreased Ki-67 proliferative indices. **C.** A significant increase in the number of apoptotic cells in the FTY720-treated group was visualized by the TUNEL assay. **D.** SPHK1 enzymatic activity in harvested tumor tissues was significantly decreased in the FTY720-treated group. The error bar represents standard error of mean. *p < 0.05, **p < 0.01.

We developed the patient-derived xenografts (PDX) model by subrenal implantation of human cervical cancer tissues. FTY720 (10 mg/kg) was injected intraperitoneally into mice every 2 days starting 1 month after the implantation of xenograft tissues and treatment was continued for 4 weeks. FTY720 significantly inhibited tumor growth in the cervical cancer PDX model (Figure 4A). We also confirmed the inhibitory effect of FTY720 on cell proliferation (Figure 4B) and its proapoptotic effect (Figure 4C). Moreover, we observed significantly decreased SPHK1 enzymatic activity in harvested xenograft tissues (Figure 4D).

## DISCUSSION

Consistent with previous reports [7, 11, 16, 22–24], this study showed that the expression of SPHK1 is increased in both cervical cancer cell lines and tissues. Of particular interest, the expression of SPHK1 was associated with well-known prognostic parameters including tumor size, invasion depth, lymph node metastasis, FIGO stage, and lymphovascular invasion. We also demonstrated that elevated expression of SPHK1 correlates with a poor prognosis and is an independent prognostic factor for predicting poor RFS. These findings implicate SPHK1 as a potentially important contributing factor in cervical cancer progression. Our results are consistent with previous studies demonstrating an association between increased SPHK1 expression and aggressive oncogenic behaviors such as larger tumor, deeper invasion depth, advanced clinical stage, poorer pathologic differentiation, higher invasive capacity, and/or chemotherapeutic resistance in head and neck cancer [25], thyroid cancer [26], salivary duct cancer [22], esophageal cancer [27], colorectal cancer [28], and bladder cancer [29]. Furthermore, previous studies have found that SPHK1 overexpression is a prognostic biomarker for the survival of patients with glioblastoma [10], head and neck cancer [6], salivary duct cancer [22], esophageal cancer [27], gastric cancer [7], and colorectal cancer [28].

We further demonstrated that blocking SPHK1 with pharmacological inhibitors significantly impaired cervical cancer cell survival and inhibited their proliferation. Moreover, treatment with FTY720 induced a significant reduction in tumor weight and proliferative activity and an increase in tumor cell apoptosis in the cervical cancer PDX model compared with controls. These findings are in agreement with our previous observations that inhibition of SPHK1 with FTY720 significantly reduced cell proliferation and increased apoptosis in ovarian cancer cells [16]. In addition, we showed that FTY720 significantly decreased the intracellular enzymatic activity of SPHK1 and the expression level of MMP-2 and VEGF-A, both of which are closely linked to tumor invasion, metastasis, and angiogenesis. These results provide new insights into the alterations of SPHK1 expression and activity that are associated with the development and progression of cervical cancer. We suggest that therapeutic targeting of SPHK1 is a potential therapeutic strategy for the treatment of cervical cancer.

Although SPHK1 and SPHK2 catalyze the same biochemical reactions, these two isoforms differ in their substrate specificities, subcellular localization, and tissue distribution [5, 30]. Several studies have shown that SPHK2 has antiproliferative effects. In contrast to the prosurvival factor SPHK1, overexpression of SPHK2 causes suppression of cell growth and cell cycle arrest [31]. Thus, SPHK1 and SPHK2 are not equivalent in their effects on biological activity despite enzymatic similarities. Strategies that have been applied to limit the effects of SPHK1-S1P signaling in cancer include inhibition of SPHK1 and/or SPHK2 and targeting of specific S1P receptors. SKI-II noncompetitively inhibits SPHK1 and SPHK2 activity. In contrast, FTY720, a S1P receptor-selective sphingosine analog, is a competitive inhibitor of SPHK1 [14]. In this study, FTY720 exerted a more potent inhibitory effect on cell survival and the production of MMP-2 and VEGF-A than SKI-II. It is possible that the antitumor effect of SKI-II via the inhibition of SPHK1 may be counteracted by its blocking effect on SPHK2, since SPHK1 and SPHK2 play opposing roles in the regulation of cell survival and apoptosis. Consequently, SKI-II is less effective than FTY720, which affects SPHK1 specifically.

In conclusion, we demonstrated that elevated expression of SPHK1 is significantly associated with the development and progression of cervical cancer. Expression of SPHK1 represents a novel and independent biomarker for the prognosis of patients with cervical cancer. Our findings indicate that inhibition of SPHK1 with pharmacological inhibitors results in potent antitumor activity in cervical cancer *in vitro* and *in vivo*. Further preclinical and clinical development of SPHK inhibitors, particularly FTY720, to treat this disease is warranted.

## MATERIALS AND METHODS

### Cell lines and treatment

Human cervical cancer cell lines (Ca Ski, HeLa, SiHa, ME-180, and MS751) were purchased from the American Type Culture Collection (Manassas, VA, USA). The cell lines were maintained in complete media (RPMI 1640 for Ca Ski; DMEM for HeLa; MEM for SiHa and MS751; McCoy’s 5A for Me-180) supplemented with 10% fetal bovine serum (FBS) and 0.1% gentamicin sulfate (Gemini Bio-Products, Calabasas, CA, USA) in 5% CO_2_ at 37°C. The SPHK inhibitors SKI-II (2-(p-hydroxyanilino)-4-(p-chlorophenyl) thiazole; Calbiochem, Merck KGaA, Darmstadt, Germany) and FTY720 (Fingolimod; Cayman Chemical, Ann Arbor, MI, USA) were resuspended in dimethyl sulfoxide at a concentration of 100 μg/mL. Cells were seeded at 3 × 10^3^ cells/well in a 96-well microplate in culture media with 10% FBS. Cells were treated with SPHK inhibitors as described in previous reports [32, 33].

### Patients and tissue specimens

A total of 287 patients with uterine cervical cancer who received surgery at the Department of Obstetrics and Gynecology at Samsung Medical Center (Seoul, Republic of Korea) between January 2002 and December 2009 were included in this study. Five normal uterine cervix specimens were used as controls. Two board-certified gynecologic pathologists examined the specimens using hematoxylin and eosin staining. Specimens in which tumor tissue accounted for more than 90% of the total volume were included in the analysis. This study was reviewed and approved by the Institutional Review Board at Samsung Medical Center (Seoul, Republic of Korea; Protocol No.: SMC # 2012-09-045).

### Western blot

Preparation of lysates from cultured cells or tumor tissues was performed as described previously [16, 34]. Protein bands were probed with a 1:1, 000 dilution of anti-SPHK1 antibody (Cell Signaling Technology, Inc., Beverly, MA, USA) or 1:3, 000 dilution of anti-β-actin antibody (Santa Cruz Biotechnology, Inc., Santa Cruz, CA, USA), and then labeled with horseradish peroxidase-conjugated anti-rabbit antibody (GE Healthcare, Piscataway, NJ, USA). Bands were visualized with an enhanced chemiluminescence kit (Amersham Biosciences, Buckinghamshire, UK) according to the manufacturer’s protocol.

### Immunohistochemistry

Immunohistochemical staining was performed on formalin-fixed, paraffin-embedded, 4-μm-thick tissue sections, using a Bond-maX automated immunostainer (Leica Biosystems, Melbourne, Australia) and a Bond Polymer Refined Detection Kit (Vision Biosystems, Melbourne, Australia). The primary antibodies were rabbit polyclonal anti-SPHK1 antibody (1:200; Cell Signaling Technology, Inc.) and mouse monoclonal anti-Ki-67 antibody (1:50; clone MIB-1, Dako, Glostrup, Denmark). Briefly, antigen retrieval was performed at 97°C for 20 min in ER1 or ER2 buffer. After blocking endogenous peroxidase activity with 3% hydrogen peroxide for 10 min, primary antibody incubation was performed for 15 min at room temperature. To verify antibody specificity, anti-mouse IgG (AI-2000; Vector Laboratories, Burlingame, CA, USA) was used instead of the primary antibody as a negative control. The degree of immunostaining of SPHK1 was evaluated according to staining proportion and intensity by combining scores for the proportion of positively stained cancer cells and the staining intensity, as previously described [11, 26]. Briefly, the area of stained cancer cells was scored as follows: 0, none; 1, <10%; 2, 10–50%; and 3, >50% of all cancer cells. The staining intensity was determined as follows: 0, no staining; 1, weak staining; 2, moderate staining; and 3, strong staining. The final score was calculated as the product of staining proportion and intensity, resulting in scores of 0, 1, 2, 3, 4, 6, and 9. The optimal cutoff value for high and low SPHK1 expression level was chosen on the basis of distribution of the staining results and a measure of heterogeneity with the log-rank test with respect to overall survival (OS). A final score ≥ 4 was used to define tumors with high SPHK1 expression, and a final score ≤ 3 was indicated low SPHK1 expression.

### 3-(4, 5-dimethylthiazol-2-yl)-2, 5-diphenyltetrazolium bromide (MTT) assay

The MTT assay was performed as previously described [34, 35]. Each sample was assayed in triplicate.

### Apoptosis assay

Cell apoptosis was measured at 48 hr using the Annexin V-FITC Apoptosis Detection Kit-1 (BD Biosciences Pharmingen, San Diego, CA, USA) according to the manufacturer’s protocol. Each sample was assayed in triplicate.

### Measurement of SPHK1 enzymatic activity

Whole cell lysates were prepared from cancer cell lines or tumor tissues as described previously [16]. The protein concentration was determined using the Bradford Protein Assay kit (Bio-Rad Laboratories, Inc., Hercules, CA, USA). Total lysate samples were assayed for SPHK1 enzymatic activity using a commercial Sphingosine Kinase Activity Assay Kit (Echelon Biosciences, Salt Lake City, UT, USA), as previously described [26].

### Enzyme-linked immunosorbent (ELISA) assay

For analysis of MMP-2 and VEGF-A secretion, culture media were harvested and centrifuged to remove cellular debris 24 hr after treatment with FTY720 or SKI-II. Media were concentrated by centrifugal filtration at 4, 000 rpm for 20 min using the Amicon Ultra-10K concentrator (Millipore, Billerica, MA, USA). ELISA kits (R&D Systems Europe, Ltd., Abingdon, UK) were used as described by the manufacturer to measure concentrations of human MMP-2 and VEGF-A. Each sample was assayed in triplicate.

### Animal care and development of *in vivo* models using patient-derived xenografts

This study was reviewed and approved by the Institutional Animal Care and Use Committee (IACUC) of the Samsung Biomedical Research Institute (Seoul, Republic of Korea; Protocol No.: H-A9-003), which is accredited by the Association for the Assessment and Accreditation of Laboratory Animal Care International and abides by the guidelines of the Institute of Laboratory Animal Resources. Tumor derived from a 32-year-old woman (Sample No. CX21) was histologically defined as FIGO stage IIA1 cervical cancer that exhibited parametrial and lymphovascular invasion. The patient received radical hysterectomy with pelvic lymph node dissection, followed by adjuvant concurrent chemotherapy and radiation therapy, and no recurrence was detected until 12 months postoperative follow-up. Female BALB/c nude mice were purchased from Orient Bio (Seongnam-si, Gyeonggi-do, Republic of Korea). For the PDX model of cervical cancer, surgically obtained tumor specimens were cut into small pieces (less than 2–3 mm) and implanted into the subrenal capsule of the left kidney of mice [36]; the tumor cells were propagated by serial transplantation. The mice used in these experiments were 6 to 8 weeks old. Mice (*n* = 10 per group) were monitored daily for tumor development and sacrificed either on day 60–70 or when any of the mice appeared moribund. We recorded body weight, tumor weight, and number of tumor nodules. Tumor tissues were fixed in 10% buffered formalin and embedded in paraffin, or placed in embedding compound (Tissue-Tek O.C.T., Sakura Finetek Japan, Tokyo, Japan) and snap-frozen with liquid nitrogen.

### Terminal deoxynucleotidyl transferase-mediated dUTP nick end labeling (TUNEL) assay

The TUNEL assay was performed as previously described [35] using the DeadEnd Fluorometric TUNEL System (Promega, Madison, WI, USA) according to the manufacturer’s instructions.

### Data analysis

The chi-square or Fisher’s exact test was performed to determine whether SPHK1 expression in cervical cancer was associated with clinicopathological characteristics. Univariate and multivariate survival analyses were used to examine the prognostic significance of SPHK1 expression in patients with cervical cancer. Curves for OS and recurrence-free survival (RFS) were generated according to the Kaplan-Meier method, and differences were analyzed using the log rank test for univariate analysis. Multivariate analysis was performed on parameters with *p* value < 0.1 in the univariate analysis using the Cox proportional hazards model (95% confidence interval) with a backward stepwise elimination method. The Mann-Whitney U test was used to evaluate significance and compare differences among groups for *in vitro* and *in vivo* assays. *p* values < 0.05 were considered to be statistically significant. The SPSS software package (version 18.0; IBM SPSS, Chicago, IL, USA) was used for all statistical analyses.
